# Resection of Nonalcoholic Steatohepatitis-Associated Hepatocellular Carcinoma: A Western Experience

**DOI:** 10.1155/2012/915128

**Published:** 2012-09-04

**Authors:** Brian Shrager, Ghalib A. Jibara, Parissa Tabrizian, Sasan Roayaie, Stephen C. Ward

**Affiliations:** ^1^Division of Surgical Oncology, Department of Surgery, Mount Sinai School of Medicine, One Gustave Levy Place, Box 1259, New York, NY 10029, USA; ^2^Department of Pathology, Mount Sinai School of Medicine, 1468 Madison Avenue, Annenberg Building, 15th Floor, Room 92, New York, NY 10029, USA

## Abstract

*Introduction*. Hepatocellular carcinoma is now known to arise in association with nonalcoholic steatohepatitis. The aim of this study is to examine the clinicopathological features of this entity using liver resection cases at a large Western center. *Methods*. We retrospectively reviewed all cases of partial liver resection for hepatocellular carcinoma over a 10-year period. We included for the purpose of this study patients with histological evidence of nonalcoholic steatohepatitis and excluded patients with other chronic liver diseases such as viral hepatitis and alcoholic liver disease. *Results*. We identified 9 cases in which malignancy developed against a parenchymal background of histologically-active nonalcoholic steatohepatitis. The median age at diagnosis was 58 (52–82) years, and 8 of the patients were male. Median body mass index was 30.2 (22.7–39.4) kg/m^2^. Hypertension was present in 77.8% of the patients and diabetes mellitus, obesity, and hyperlipidemia in 66.7%, respectively. The background liver parenchyma was noncirrhotic in 44% of the cases. Average tumor diameter was 7.0 ± 4.8 cm. Three-fourths of the patients developed recurrence within two years of resection, and 5-year survival was 44%. *Conclusion*. Hepatocellular carcinoma may arise in the context of nonalcoholic steatohepatitis, often before cirrhosis has developed. Locally advanced tumors are typical, and long-term failure rate following resection is high.

## 1. Introduction

Nonalcoholic fatty liver disease (NAFLD) is the most prevalent form of chronic liver disease in the West today [1]. NAFLD is associated with the diseases of diabetes mellitus (DM) and obesity and has come to be considered a hepatic manifestation of the metabolic syndrome [2–4]. The most severe form of NAFLD is an inflammatory and fibrosing parenchymal lesion known as nonalcoholic steatohepatitis (NASH) [5]. NASH affects roughly 3% of adults in Western countries [1] and approximately 25–30% of the morbidly obese [6]. A subset of NASH patients will ultimately develop frank cirrhosis, with its potential end-points of liver failure and hepatocellular carcinoma (HCC) [7].

 DM and obesity have each been shown to increase risk for liver cancer occurrence or liver cancer-related mortality in large-scale prospective cohort studies [8–11]. It is logical to assume that NASH is the connecting link between these metabolic diseases and liver malignancy. Surprisingly, however, few clinical studies have been devoted to investigating NASH-associated HCC [1, 12–14] (Table 1). Similarly, few surgical series have been published [15–19] (Table 2). Of note, all papers describing partial liver resection for NASH-HCC originate from Japan. In this study, we report a Western series of liver resections for HCCs arising in histologically-active NASH. The aims of the study are to (a) examine the clinical and pathological features of NASH-associated HCC using the accuracy of surgical specimens and to (b) evaluate long-term survival and recurrence outcomes following curative treatment.

## 2. Materials and Methods

### 2.1. Inclusion Criteria

After obtaining consent from an institutional review board, prospectively collected data on all patients receiving partial liver resection for nonfibrolamellar HCC at Mount Sinai Medical Center from January 2000 to December 2009 was reviewed. The purpose of this paper was to identify all patients with tumors arising against a background of histologically-active NASH. 

 NASH was defined by the following criteria: (a) histological evidence in the nonneoplastic liver parenchyma of steatosis with varying degrees of ballooning hepatocytes, Mallory bodies, lobular inflammatory infiltrate, and fibrosis [5]; (b) an absence of clinically significant alcohol intake (less than 20 gm/day of ethanol consumption) and no personal history of alcoholism; (c) negative serology for Hepatitis B surface antigen; (d) negative serology for anti-Hepatitis C virus antibody and/or no evidence of Hepatitis C viral RNA on polymerase chain reaction (PCR), and (e) no histological, serological, chemical and/or clinical evidence for other parenchymal liver diseases including, but not limited to, autoimmune hepatitis, primary biliary cirrhosis, hereditary hemochromatosis, Wilson's disease and drug-induced liver injury. For a cirrhotic patient to be included into the study, some degree of residual NASH histology was required. 

### 2.2. Definitions

DM was defined as a fasting blood glucose ≥126 mg/dL on two occasions or current treatment with insulin or oral hypoglycemic agent(s) [20]. Hypertension (HTN) was defined as a resting blood pressure of ≥140/90 mmHg on two separate occasions or current treatment with antihypertensive medication(s). Hyperlipidemia was defined as total serum cholesterol ≥220 mg/dL or serum triglyceride ≥150 mg/dL on two separate occasions or current treatment with lipid-lowering medication(s) [21]. Obesity was classified by a body mass index (BMI) >28.8 kg/m^2^ [22].

### 2.3. Diagnosis and Treatment of HCC

Diagnosis was established using contrast-enhanced CT scan of the chest and abdomen ± MRI of the abdomen as per the radiographic criteria laid out by the European Association for the Study of the Liver [23]. A patient was deemed resectable if synthetic and excretory liver functions were preserved (Child-Pugh class A liver function) and radiographic/hematologic stigmata of portal hypertension were absent. 

Following resection, patients were followed with clinical, laboratory, and radiographic assessment every 3 months for the first year, every 4 months for the second year, and bi-annually thereafter. Patients with a solitary liver recurrence and Child-Pugh A liver disease and no evidence of portal hypertension underwent a second hepatic resection. Patients with multiple intrahepatic recurrences or compromised hepatic function were treated with radiofrequency ablation (RFA) and/or transarterial chemoembolization (TACE). Patients with recurrence confined to the liver and without significant comorbidities were also referred for liver transplantation. Patients ultimately receiving liver transplant were censored on the date of transplant. After 2007, patients not eligible for repeat resection, liver transplantation, or local-regional therapies were treated with sorafenib.

### 2.4. Pathological Analysis

Specimens were independently reviewed by two attending pathologists. Specimens were routinely fixed in hemotoxylin and eosin stain, Masson's trichrome stain for collagen fibers, and Prussian blue stain for iron granules. Additional specialized stains were used on a selective basis. Tumor size was measured at the widest diameter of the dominant nodule. Satellite nodules were defined as tumors ≤2 cm in diameter and within 2 cm of the dominant nodule. *“By contrast, additional HCC implants outside of the satellite criteria, that is, >2 cm in diameter or >2 cm away from the index tumor, defined multinodularity.”* Tumor grade was reported as well, moderate or poorly differentiated using the Edmonson classification [24]. The Brunt criteria were used to quantify the degree of hepatic steatosis and to grade the level of lobular inflammatory activity [5]. Fibrosis was also staged according to the descriptions of Brunt et al. [5]: F0, no fibrosis; F1, pericellular and/or perivenular fibrosis confined to Zone 3; F2, pericellular fibrosis extending to Zones 2 and 3 with or without portal fibrosis; F3, bridging fibrosis; and F4, cirrhosis. For the purpose of this study, F0 to F2 fibrosis was considered “noncirrhotic.”

## 3. Results

### 3.1. Patients

Of the 548 patients undergoing partial liver resection for HCC, 255 (46.5%) showed serological evidence for Hepatitis B viral (HBV) infection, 178 (32.5%) for Hepatitis C viral (HCV) infection, and 5 (0.9%) for coinfection with both viruses. 20 patients (3.6%) displayed alcoholic liver disease. 7 patients (1.3%) had hereditary hemochromatosis. 2 patients had *α*-1 antitrypsin deficiency, 2 had primary biliary cirrhosis, and one had Gaucher's disease. 18 patients (3.3%) had cryptogenic cirrhosis (CC) and 51 patients (9.3%) had noncirrhotic livers without evidence for underlying parenchymal liver disease despite comprehensive investigation. The remaining 9 patients (1.6%) developed their tumors in association with histologically-active NASH; these cases form our series. 

**Figure 1:**
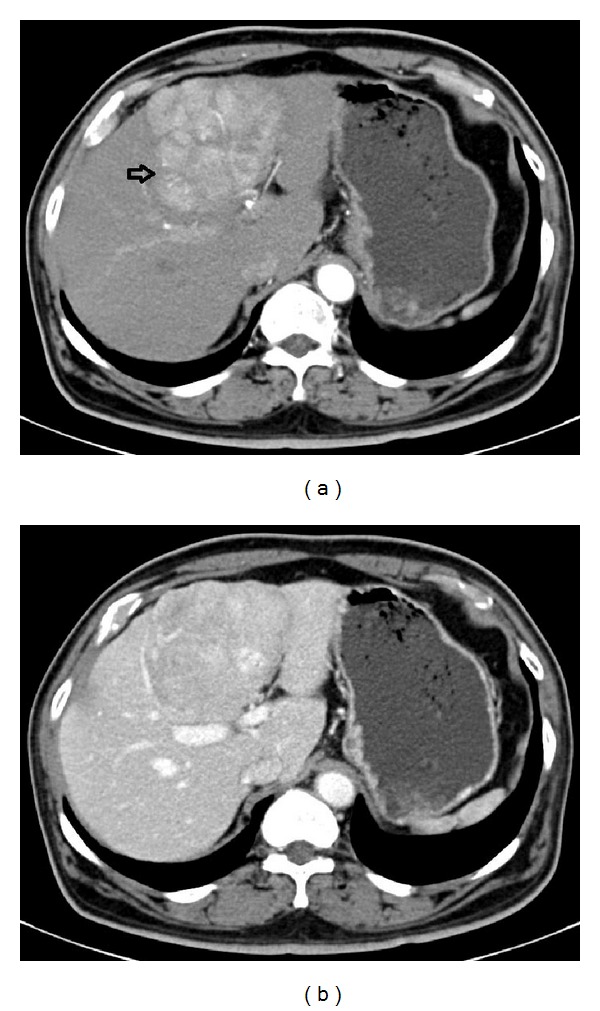
Contrast-enhanced CT scan of NASH-associated HCC. (a) This arterial-phase image shows an enhancing lesion (arrow) in the left lobe of a steatotic liver. No gross radiographic evidence of cirrhosis is present. (b) The same tumor shows hypoenhancement or “washout” in the delayed portal venous-phase image.

 Of the 9 patients with NASH-associated HCC, 2 presented with tumor-related symptoms, specifically abdominal pain and weight loss; the remaining 7 cases were discovered incidentally. HCC diagnosis was established radiographically in each case (Figure 1), with tissue biopsy acting as a supplement in 6 of the 9 cases. 

 Median age at diagnosis was 58 (52–82) years, and 8 of the patients were male (Table 3). Median BMI was 30.2 (22.7–39.4) kg/m^2^. All of the patients carried a diagnosis of at least one metabolic disease, with HTN displaying the highest prevalence (77.8%). Obesity, DM, and hyperlipidemia each displayed a prevalence of 66.7%, respectively. In only one case was serum *α*-fetoprotein elevated above 200 ng/mL. All patients demonstrated Child-Pugh class A liver function.

### 3.2. Pathology

**Figure 2:**
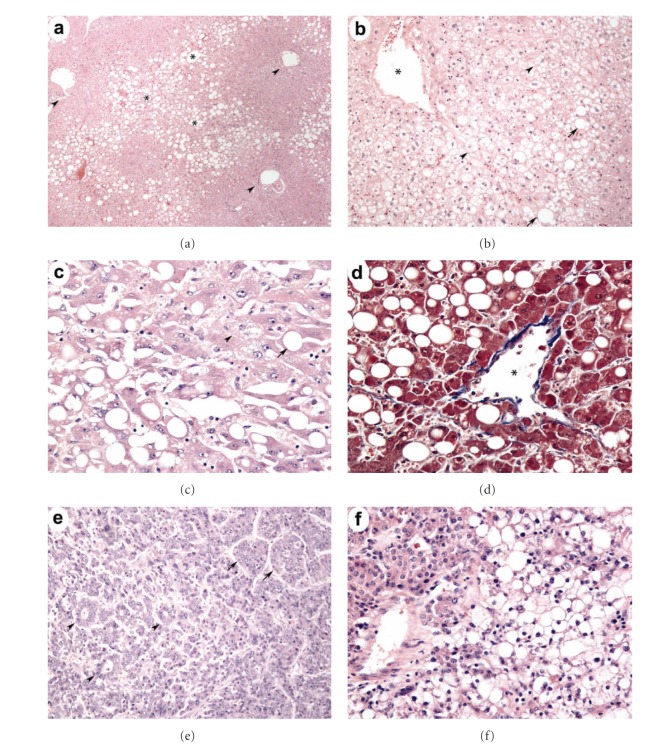
Histopathological features of noncirrhotic NASH-associated HCC. (a) The nonneoplastic liver shows macrovesicular steatosis involving the perivenular zone (∗) with sparing of the periportal zones (arrowheads) (patient 8, H&E stain, 40x). (b) and (c) show at greater magnification the steatotic hepatocytes (arrows) and ballooning degeneration of hepatocytes (arrowheads) evident in the perivenular zone of the nonneoplastic parenchyma (patients 4 and 7, H&E stain, 100x and 200x). (d) Specialized collagen staining shows the central venule (∗) to be invested with a band of perivenular fibrosis; bridging fibrosis and cirrhotic nodules are notably absent (patient 7, Masson's trichrome stain, 200x). (e) and (f) show two examples of HCC arising within noncirrhotic NASH showing pseudoglandular (arrowheads) and trabecular (arrows) features and fat within tumor cells (right frame) (patients 7 and 8, H&E stain, 100x).

One resection was noncurative with a positive microscopic surgical margin. Another patient had intraoperative evidence of metastatic disease in the greater omentum that was completely excised. Mean tumor size was 7.0 ± 4.8 cm (Table 4). One patient had a multinodular HCC. Gross vascular invasion was present in 2 cases and microscopic vascular invasion in 4. All tumors except one were well or moderately differentiated, and 4 tumors showed varying degrees of steatosis within neoplastic cells. Of interest, four of the cases (44.4%) showed a noncirrhotic parenchyma (all F1).  Figure 2 shows salient histopathological findings from three of these noncirrhotic cases. In accordance with the inclusion criteria of this study, all cases displayed the histological hallmarks of NASH in the background liver. 

### 3.3. Long-Term Outcome

Median length of followup was 38.3 (1.1–105.2) months. One patient died of sepsis 33 days after surgery. Of the remaining 8 patients, 7 recurred (87.5%). The median time to recurrence was 8.3 (6.2–37.9) months and 6 of the 7 patients that recurred did so within 2 years of the index resection. Initial recurrence was limited to the liver in 6 of 7 cases. One patient received a liver transplant 6.4 months following index resection and is still alive at the time of this report. Median survival was 38.3 (1.1–105.2) months. 5-year survival was 44.4%. Detailed long-term outcome data is provided in Table 4. 

## 4. Discussion

It has become widely accepted that NASH is a hepatic manifestation of the metabolic syndrome. The heavy prevalence of DM, HTN, obesity, and hyperlipidemia in our series gives further clinical support to this concept. Moreover, our study demonstrates the sclerotic spectrum of NASH, with varying degrees of fibrosis seen originating from the centrilobular zone. 

Our study consisted of patients with a large mean tumor size of 7.0 cm. The finding of more advanced tumors in our series is related to the modes of presentation; all cases were either discovered incidentally or because of mass-related symptoms. None of the patients had been enrolled in radiographic surveillance programs for HCC. Similarly, Giannini et al. found that CC-associated HCCs were more likely to be discovered at an advanced stage and less likely to be amenable to treatment when compared to HCV-associated HCCs; this was also attributed to less surveillance among the former group [25]. It follows that of our 8 patients that survived the postoperative period 6 recurred within two years of resection, a clear sequella of the advanced nature of their initial tumors. Despite this high early recurrence rate, we were able to achieve a 5-year survival of 44%, likely attributable to the aggressive multimodality approach to treating those recurrences. 

### 4.1. Fibrosis: A Necessary Precursor?

NASH is felt to progress to cirrhosis in 3–15% of cases [7], and it has been suggested that the development of cirrhosis is a necessary intermediate step in a progression to HCC [6]. This has been a difficult theory to prove, in part due to the disappearance of the histopathological features of NASH once cirrhosis is established [15, 26]. Nevertheless, “cryptogenic cirrhosis” has been shown to likely represent end-stage NASH based on clinical parameters [3, 27], with a risk of HCC development that rivals HCV-associated cirrhosis [7]. In a large case-control study, Hashimoto et al. showed that the strongest independent predictor for HCC development in NASH patients was severe hepatic fibrosis [14].

 Despite this evidence, 44% of our patients showed only mild fibrosis in the nonneoplastic liver parenchyma. Similar findings have been echoed in other surgical series. Hashizume et al. found that 3 of 8 patients undergoing curative treatments (6 resections, 2 RFAs) of NASH-associated HCC had noncirrhotic livers [15]. Kawada et al. found that 5 of 8 patients receiving resection of NASH-associated HCC showed only mild fibrosis (F2) in their background livers [16]. Paradis et al. analyzed a group of 31 patients receiving resection of HCC that complicated only metabolic syndrome (81% with some form of NAFLD) and found nonfibrotic or mildly fibrotic livers in 20 (65.5%) of the cases [28]. 

 It is important to point out the natural selection bias for noncirrhotics that exists in a surgical resection series such as ours. Further supporting data, however, exists in nonsurgical studies [1, 29, 30]. Guzman et al. found from a cohort of 50 HCC patients submitted to a wide spectrum of treatments 3 of 5 NAFLD-associated cases that were noncirrhotic [30]. In a larger series, Ertle et al. showed that in a group of 36 NASH-associated HCC patients of which only a minority received resection, the prevalence of noncirrhotic background liver was 47.2% [1]. 

 We chose not to include cases of cryptogenic cirrhosis (CC) in our study group and instead included only patients with histologically-active NASH. Accepting the premise that the 18 CC cases in our entire population represented “burnt-out” NASH, the actual proportion of NASH-associated HCC cases that developed in the absence of cirrhosis becomes 14.8%. This is similar to the rate of noncirrhotic HCV-associated HCCs in our overall cohort (21/178, 11.8%, *P* = 0.751 by Fisher's exact test); it is less but also statistically similar to our rate of noncirrhotic HBV-associated HCCs (68/255, 26.7%, *P* = 0.246 by Fisher's exact test). Indeed, based on our experience and that of other investigators, the relevance of this entity should not be underestimated. 

 If the carcinogenic milieu of a cirrhotic liver represents only part of the story, the additional mechanisms underlying HCC development in the NASH liver have yet to be fully elucidated. Recent investigation has centered on the oncogenic effects of hyperinsulinemia, a key component of the metabolic syndrome [31–33]. Additional research has focused on the oxidative stress present in the microenvironment of the steatotic liver. Specifically, lipid peroxidation, an important component of disease progression in NASH, has been implicated in the generation of reactive oxygen species that may possess mutagenic qualities sufficient to initiate malignant transformation [6, 34, 35]. An additional effect of this oxidative stress has been shown to include clonal expansion of premalignant oval cells in both mouse and human forms of fatty liver [35–37]. Further proliferation of these neoplastic cells may be driven by disturbances in cytokines and growth factors [6, 38]. Whether HCC development in NASH is an effect of factors directly derived from the underlying metabolic diseases or a result of biochemical derangements in the steatotic liver lesions is a question which remains to be answered. 

 “While the potential mutagenicity of the noncirrhotic NASH parenchyma is intriguing, we must acknowledge that alternate etiological agents might have been at play in these patients. One possibility is occult HBV infection, as evidenced by the presence of HBV DNA by PCR analysis in the context of a negative serological panel. Unfortunately, only 1 of the 4 noncirrhotic patients in our series received this PCR analysis (negative), and this scenario cannot be ruled out in the other three. Environmental exposure to a hepatocarcinogen such as aflatoxin A, nitrosamine, or benzopyrene serves as an additional plausible, albeit unlikely, etiology. Finally, advanced age (one noncirrhotic over 70) and male gender (all 4 noncirrhotics) placed these patients at slightly increased risk of primary hepatic malignancy.” 

In conclusion, HCC may arise in a liver affected by NASH, often in association with multiple metabolic comorbidities. Although cirrhosis increases the risk of malignant transformation, it does not appear to be a necessary precursor to such an event. NASH-HCC often presents at a late stage leading to increased local failure following resection; nevertheless, with an aggressive approach to recurrence, long-term survival may still be achieved. With the increasing prevalence of obesity and diabetes mellitus in Western populations, investigation into the utility of HCC surveillance for patients with established NASH seems warranted. 

## Figures and Tables

**Table 1:** Series of NASH-associated HCCs (≥5 patients).

	*n*	Age (years)*	Gender (M : F)	Comorbidities	Cirrhosis (%)
Shimada et al. [12]	6	65.7	3 : 3	Ob 50%, DM 50%, HL 17%	100
Chagas et al. [13]	7	63	4 : 3	Ob 43%, DM 57%, HL 29%	85.7
Hashimoto et al. [14]	34	70 (median)	21 : 13	Ob 62%, DM 74%, HL 29%, HTN 47%	88
Ertle et al. [1]	36	68.6	32 : 4	Ob 95%, DM 64%, HL~50%, HTN > 70%	52.8

Ob: obesity, DM: diabetes mellitus, HL: hyperlipidemia, HTN: hypertension.

*Expressed as a mean unless otherwise indicated.

**Table 2:** Surgical series of NASH-associated HCCs (≥5 patients).

	*n*	Age (years)	Gender (M : F)	Tumor size (cm)	Primary Tx	Cirrhosis (%)	Recurrence (%)
Hashizume et al. [15]	9	71.5**	6 : 3	3.8*	LR 67%, RFA 22%, TAE 11%	67	11.1
Kawada et al. [16]	6	73**	3 : 3	3.5**	LR 100%	0	N.R.
Malik et al. [17]	17	63.1*	12 : 5	3.4*	LT 100%	100	5.9
Tokushige et al. [18]	16	N.R.	N.R.	N.R.	LR 81%, RFA 18%	N.R.	88
Takuma and Nouso [19]	11	73.8*	6 : 5	3.3*	LR 64%, RFA 27%, MCT 9%	36	60^a^

LR: liver resection, RFA: radiofrequency ablation, TAE: transarterial embolization, LT: liver transplant, MCT: microwave coagulation therapy.

*Mean, **median, ^a^recurrence-free survival.

**Table 3:** Demographic and clinical data.

Patient	1	2	3	4	5	6	7	8	9
Age gg	58	57	73	52	78	82	52	57	73
Gender	M	M	F	M	M	M	M	M	M
BMI (kg/m^2^)	29.1	30.2	26.4	39.4	32.2	27.4	31.1	34.6	22.7
Metabolic disease	HTN	DM	DM	DM HTN HL	DM HTN HL	HTN HL	HTN HL	DM HTN HL	DM HTN HL
Symptoms	No	No	No	No	No	No	Yes	No	Yes
Biopsy	No	No	Yes	Yes	Yes	Yes	No	Yes	Yes
AFP (ng/mL)	7.1	3.1	100.2	2.1	N.A.	7.4	311,190	9.3	2.5
ALT (IU/L)	60	44	52	N.A.	106	35	49	235	23
Albumin (g/dL)	4.3	3.9	4.2	4.5	3.6	4.3	3.6	3.3	3.7
Bilirubin (mg/dL)	1.1	1.0	0.8	0.3	1.3	1.0	0.8	0.5	0.5
Creatinine (mg/dL)	0.7	1.3	1.2	1.0	0.7	1.1	0.8	0.9	0.9
Platelet (×1000/*μ*L)	133	65	112	362	340	154	590	218	316
INR	1.0	1.3	0.9	1.0	1.1	1.0	1.1	0.9	1.1
CTP class	A	A	A	A	A	A	A	A	A
MELD score	6.6	9.0	7.2	7.3	6.4	6.8	6.9	6.4	6.9

BMI: body mass index, DM: diabetes mellitus, HTN: hypertension, HL: hyperlipidemia, AFP: alpha-fetoprotein, ALT: alanine aminotransferase, INR: international normalization ratio, CTP: Child-Turcotte-Pugh, MELD: model for end stage liver disease, N.A.: data not available.

**Table 4:** Pathological data and longterm outcomes.

Patient	1	2	3	4	5	6	7	8	9
Tumor size (cm)	5.1	1.8	6.2	6.0	4.5	6.3	19.0	6.0	8.4
Number of nodules	1	1	1	1	2	1	1	1	1
Vascular invasion	None	None	Micro	None	Gross	Micro	Gross	Micro	Micro
Satellites	Yes	No	Yes	No	No	No	Yes	No	No
Tumor cell differentiation^a^	Well	Well	Well	Mod	Mod	Mod	Poor	Mod	Mod
Steatotic tumor cells	No	Yes	Yes	No	No	No	No	Yes	Yes
Parenchymal fibrosis^b^	F4	F4	F4	F1	F3	F3	F1	F1	F1
Margin (mm)	3	5	0	20	10	10	10	2	2
Regional lymph nodes (+)	No	No	No	No	No	No	No	No	No
Metastases	No	No	No	No	No	No	Yes	No	No
Recurrence gg	Yes	Yes	Yes	No	Yes	Yes	No	Yes	Yes
Time to recurrence (months)	19.4	6.4	8.3	—	6.2	37.9	—	6.2	16.8
Distribution of 1st recurrence	IH	IH	IH	—	IH	IH	—	IH/EH	IH
Treatment of 1st recurrence	TACE	OLT	TACE	—	RR	RFA	—	RR	RFA
Survival (months)	105.2	32.3	27.8	84.0	62.1	68.0	1.1	34.7	38.3

Micro: microscopic, Mod: moderate, IH: intrahepatic, EH: extrahepatic, TACE: transarterial chemoembolization, OLT: orthotopic liver transplant, RR: repeat resection, RFA: radiofrequency ablation.

^a^Based on Edmonson grading system, ^b^based on Brunt criteria.
